# Parkinson Symptom Severity and Use of Nutraceuticals

**DOI:** 10.3390/nu15040802

**Published:** 2023-02-04

**Authors:** Laurie K. Mischley, Joshua Farahnik, Ludwig Mantay, Jamie Punzi, Kayla Szampruch, Tyrice Ferguson, Devon J. Fox

**Affiliations:** 1 Parkinson Center for Pragmatic Research, Shoreline, WA 98133, USA; 2 Bastyr University Research Institute, Bastyr University, Kenmore, WA 98028, USA

**Keywords:** nutraceutical, functional food, neuroprotection, natural compounds, Parkinson’s Disease, supplement, population, epidemiology

## Abstract

Background: It is estimated that half of the individuals with Parkinson’s disease (PD) use some form of over-the-counter vitamin, herbal supplement or nutraceutical. The goal of this study was to survey individuals with PD about their use of the nutraceuticals and evaluate the association of the nutraceutical with the severity of symptoms. Methods: Participants with self-reported idiopathic PD within the 2021 cohort (*n* = 1084) were included in a cross-sectional study to assess association of nutraceuticals with symptom severity via linear regression analysis. PD severity was measured using the patient-reported outcomes in PD, and supplement use reflected self-reported consistent use over the previous six months. All regression analyses adjusted for age, gender, income and years since diagnosis. The use of the term progression refers to PRO-PD scores adjusted for years since diagnosis. Results: The most frequently used supplements were vitamin D (71%), B12 (44%), vitamin C (38%) and fish oil (38%). None of the supplements being used were associated with statistically significant worse outcomes. Nutraceuticals associated with improved outcomes were Ginkgo biloba (GB), NAD+ or its precursors, 5-methyltetrahydrofolate, glutathione, mucuna, CoQ10, low dose lithium, curcumin, homocysteine factors, DHEA, coconut oil, vitamin C, and omega-3 fatty acids (fish oil). Conclusions: These data suggest that in a real-world setting, some over-the-counter supplements are associated with fewer patient-reported symptoms. Supplements with significant associations with fewer symptoms have biological plausibility and future clinical trials should be explored.

## 1. Introduction

Nutraceuticals are concentrated sources of minerals, molecules and plant extracts taken with the intention of providing a medical or health benefit [1,2,3]. Nutraceuticals are growing in popularity with an annual growth rate of 9% and the global industry is projected to reach over $300 billion by 2030 [4], largely motivated by personalized and preventative medicine trends [5] and reduced nutrients from food sources [6,7]. Studies show that approximately half of the population is taking at least one supplement [8], and patients with PD estimates of use are reported to be almost 60% [9].

Based on biological plausibility and pre-clinical evidence of potential benefit [10,11,12], several dietary supplements have been formally studied in PD clinical trials, such as coenzyme Q10 [13,14,15], creatine [16,17], glutathione [18,19,20], *N*-acetyl cysteine (NAC) [21] and nicotinamide [22]. The results have been mixed in randomized clinical trials, suggesting either true non-efficacy of nutraceuticals or that our pre-clinical models, traditional outcome measures, and/or study designs are flawed. A subjective outcome measure capable of capturing and quantifying non-motor symptoms may be more useful early in the disease, and for detecting subtle shifts in metabolic function.

The patient-reported outcomes in Parkinson’s Disease (PRO-PD) scale is an entirely subjective outcome measure initially created for use in the modifiable variables in Parkinsonism (MVP) study, a prospective observational study designed to identify lifestyle factors associated with PD progression. In a 2017 cross-sectional analysis, the MVP study cohort (*n* = 1053) showed that coenzyme Q10 (*p* = 0.026) and fish oil (*p* = 0.019) were associated with improved outcomes, and iron supplementation was associated with faster PD progression (*p* = 0.022) [23]. Because trends in supplement use change over time, the goal of this study was to describe the nutraceuticals and over-the-counter (OTC) dietary supplements most frequently used among the 2021 MVP study participants and conduct an updated analysis to evaluate whether use of any supplements were associated with PD symptom severity. The aim of this study was to use the pragmatic, real-world study design of the MVP study to evaluate the association of nutraceutical use on a subjective outcome measure (PRO-PD). 

## 2. Materials and Methods

The MVP study is a prospective, internet-based natural history study (*n* > 2900). Individuals were recruited via Facebook, YouTube, and other social media outlets, ClinicalTrials.gov, MJFF Trial Finder, Washington State PD Registry, and at lectures and events related to PD wellness. The study was approved by Bastyr University IRB [IRB #11A-1301; Clinical Trials # NCT02194816] and a waiver of documentation of informed consent was issued for this project. All individuals acknowledged that they read and understood the participant information sheet before answering the study questions. Individuals are asked to fill out surveys of approximately 60–90 min twice per year, and while each survey has a consistent set of questions, additional questions intended to inquire about specific topics are added and removed with each iteration. Participants do not need to complete the survey in one sitting. Only survey responses between February and December of 2021 were included in this analysis. Only individuals reporting an idiopathic Parkinson’s disease diagnosis were included; those with other forms of parkinsonism were eliminated from the dataset. For individuals who completed the survey twice within the selected timeframe, the most recent survey response was kept, and any prior submissions were removed. 

To measure supplement use, participants were given a list of commonly used supplements among people with PD, as determined by a literature review, clinical experience, patient feedback, and academic expertise in nutrition and neurological health among the study team. The list has expanded over time to include new supplements as trends change, and new products are brought to the attention of study staff. Participants were asked to indicate all the supplements they had taken consistently (Y/N) over the prior six months. Only supplements used by five or more individuals were included in this analysis. 

The patient-reported outcomes in PD (PRO-PD) was the primary outcome measure. The PRO-PD is a series of 33 slider bars, each ranging from 0 (asymptomatic) to 100 (severe), and each representing a common PD symptom. The total PRO-PD is the unweighted cumulative score of all variables. According to prior publications from this dataset, the mean increase in the PRO-PD total score is 38 (±14) points per year. The PRO-PD has been shown to be correlated with legacy measures, including the PDQ-39 (r = 0.763, *p* < 0.000), PROMIS Global quality of life (r = −0.7293, *p* < 0.000), Hoehn & Yahr (HY) (r = 0.5922, *p* <0.000), total unified PD rating scale (r = 0.4724, *p* < 0.000), and the timed-up-&-go (r = 0.4709, *p* < 0.000) [24]. 

Multiple linear regression models were used to examine the association between nutraceutical use and overall PD severity, with PRO-PD scores used as the outcome variable. Regression analyses controlled for age, years since diagnosis, income and gender. For regression analyses, participant records were excluded if age, years since diagnosis, income and/or gender data were missing. The use of the term ‘progression’ throughout this manuscript refers to PRO-PD scores adjusted for years since diagnosis. All variables used in the PRO-PD score were required to be answered, therefore there were no missing values. For QoL analysis using PROMIS, only complete data were included, and no substitutions were made. Study data were collected and managed using Research Electronic Data Capture REDCap hosted by Bastyr University. REDCap is a secure, web-based application supporting data capture for research which provides an intuitive interface for validated data entry, data manipulation and export procedures, automated export procedures and procedures for importing data from external sources [25,26].

All statistical analyses were performed using STATA Version 14 (College Station, TX, USA) with alpha set to 0.05. To avoid increasing the risk of type II errors or false negatives, no adjustments were made for multiple comparisons, as this study was designed to screen for possible associations. 

## 3. Results

Of the 1084 individuals that met inclusion criteria, 92% identified as Caucasian, 55% were women, 47% had graduate or professional degrees, and 19% reported an income greater than $150,000 US dollars per year. Whereas PD affects more men than women in the population, this study cohort was predominantly female (54.7%). On average, participants reported 7.8 years since diagnosis and 41% reported one-sided symptoms only (Table 1). A total of 975 (89.9%) participants reported using at least one supplement and among people who used supplements, the average supplement-user reported using five distinct supplements over the past six months, with a range from 1 to 28 supplements per day.

**Figure 1:**
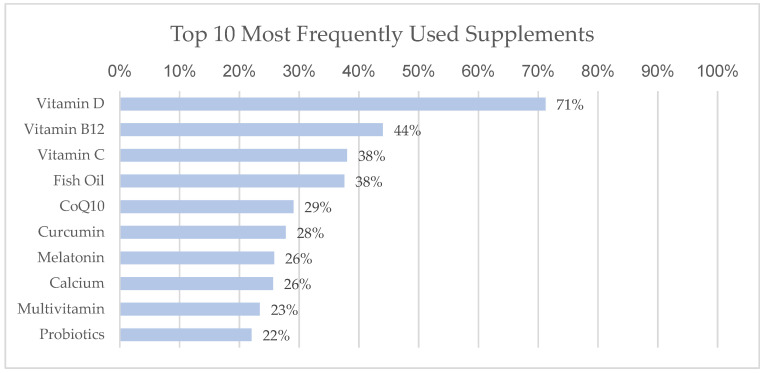
Ten most used supplements in this sample cohort with percentage of cohort using the supplement.

Of the 43 supplements that were on the list, 3 were dropped because they were being used by less than five participants, 13 (30%) were associated with fewer symptoms over time, none were significantly associated with more symptoms over time, and 31 (70%) were associated with no statistically significant difference in outcomes. The most commonly used supplements were vitamin D (*n* = 772, 71%), vitamin B12 (*n* = 477, 44%), vitamin C (*n* = 412, 38%) and fish oil (*n* = 407, 38%) (Figure 1).

**Figure 2:**
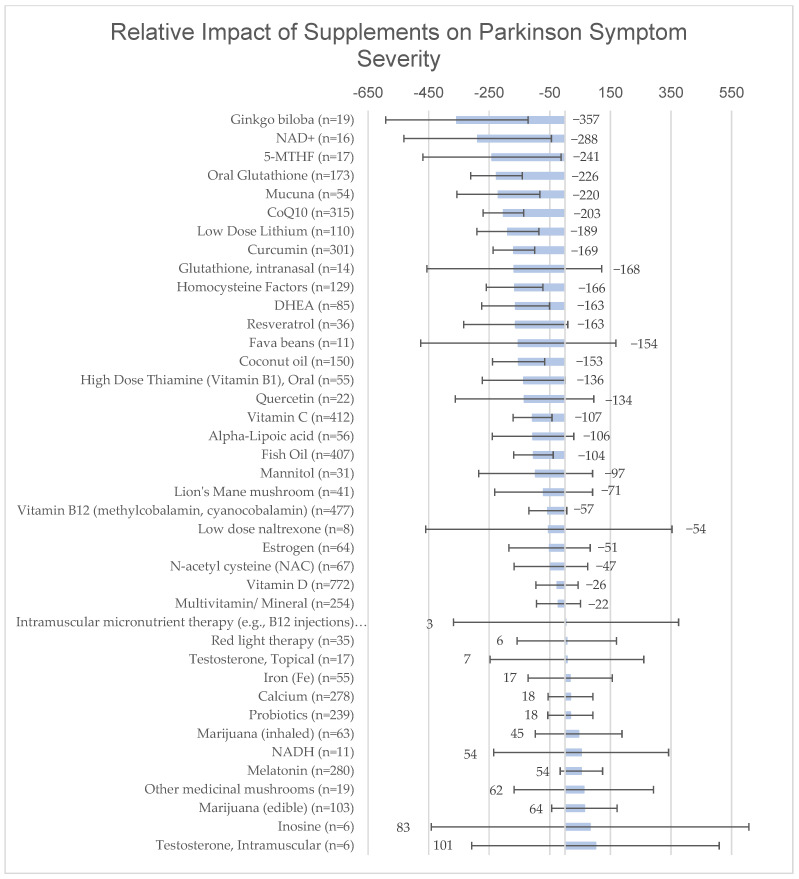
Relative impact on PRO-PD scores with 95% confidence intervals by linear regression adjusted for age, gender, income and years since diagnosis.

After adjusting for age, gender, income and years since diagnosis, individuals reporting consistent use of the following supplements reported fewer PD symptoms statistically over time: Ginkgo biloba (β = −357, CI95%: −592–−122), NAD+ (β = −288, CI95%: −532- −45), 5-methyltetrahydrofolate (5-MTHF) (β = −241, CI95%: −469–−13), oral glutathione (β = −226, CI95%: −311–−141), mucuna (β = −220, CI95%: −257–−83), coenzyme Q10 (β = −203, CI95%: −270–−136), low-dose lithium (β = −189, CI95%: −291–−86), curcumin/turmeric (β = −169, CI95%: −237–−100), homocysteine-lowering b-vitamin complex (e.g., vitamin B6, B12, folate, betaine) (β = −166, CI95%: −260–−73), dehydroepiandrosterone (DHEA) (β = −163, CI95%: −275–−51), coconut oil (β = −153, CI95%: −239–−67), vitamin C (β = −107, CI95%: −171–−43), and fish oil (β = −104, CI95%: −169–−39), (all *p* < 0.05) (Figure 2).

Supplements not significantly associated with a statistically significant difference in outcomes included: alpha-lipoic acid (β = −106, CI95%: −240–28.5; *p* = 0.1), vitamin B12 (β = −56.5, CI95%: −119–6.02; *p* = 0.08), calcium (β = 18.13, CI95%: −56.2–92.5; *p* = 0.6), vitamin D (β = −26.5, CI95%: −96.4–43.3; *p* = 0.5), estrogen (β = −51.0, CI95%: −185–82.9; *p* = 0.5), NADH (β = 53.5, CI95%: −235–342; *p* = 0.7), intranasal glutathione (β = −168, CI95%: −456–121; *p* = 0.3), inosine (β = 82.8, CI95%: −442–607; *p* = 0.8), iron (β = 17.1, CI95%: −122–156; *p* = 0.8), edible marijuana (β = 64.0, CI95%: −43.7–172; *p* = 0.2), inhaled marijuana (β = 45.0, CI95%: −98.0–188; *p* = 0.5), melatonin (β = 53.7, CI95%: −16.2–123; *p* = 0.1), probiotics (β = 17.7, CI95%: −56.6- 92.0; *p* = 0.6), quercetin (β = −134, CI95%: −362–94.6; *p* = 0.3), resveratrol (β = −163, CI95%: −334–8.52; *p* = 0.06), multivitamin/multimineral supplement (β = −21.8, CI95%: −94.2- 50.6; *p* = 0.6), *N*-acetyl cysteine (β = −46.7, CI95%: −168–74.8; *p* = 0.5), low-dose naltrexone (β = −53.5, CI95%: −460–353; *p* = 0.8), fava bean (β = −154, CI95%: −476–168; *p* = 0.3), high dose thiamine (oral) (β = −136, CI95%: −273–1.20; *p* = 0.052), lion’s mane mushroom (β = −70.5, CI95%: −232–91.0; *p* = 0.4), other medicinal mushrooms (β = −61.8, CI95%: −168–292; *p* = 0.6), testosterone (intramuscular, β = 101, CI95%: −308–509; *p* = 0.6; and topical, β = −6.53, CI95%: −247- 260; *p* = 0.9), mannitol (β = −97.3, CI95%: −285- 90.8; *p* = 0.3), IV micronutrient therapy (β = −419, CI95%: −1330–487; *p* = 0.4), IM micronutrient therapy (β = −57.6, CI95%: −75.2–40.0; *p* = 0.9), red light therapy (β = 6.03, CI95%: −158–170; *p* = 0.9), and farnesol (β = −235, CI95%: −207–754; *p* = 0.6). 

## 4. Discussion

This cohort study demonstrated that supplementation with certain nutraceuticals was associated with reduced patient-reported PD symptom severity over time. The cross-sectional nature of this study does not allow for causal inference and in addition, confounders permeate the data. It is possible that individuals supplementing with nutraceuticals may also be more likely to afford gym memberships, fresh organic food, and more specialized health care providers and better pharmaceuticals for symptom management, in which case supplement use may only be a surrogate. Routine use of nutraceuticals may induce a placebo response and while this dopamine-mediated placebo response may contribute to the effectiveness of a medication, it would be expected that all supplements would offer some baseline improvement, which was not observed here. 

This study generally demonstrates safety in nutraceuticals. While a number of supplements were associated with higher PRO-PD scores over time, none of these associations reached statistical significance. The previous iteration of this study conducted on data from 2017 showed supplementation with iron and melatonin significantly increased symptom severity, however these were not significant in this updated analysis [23]. Differences may be in part, due to changes in participants or confounders such as exercise [27], cognition, mental health status, or other psychosocial factors [28]. Cross-sectional analyses introduce year to year changes that are largely unable to be accounted for. 

### Strengths and Limitations

Strengths of this study include the large sample size (*n* = 1083), an updated and streamlined questionnaire that systemically queried participants about a diverse list of supplements, and relevance of topic in the growing supplement industry. With respect to the Bradford-Hill criteria for determining causation, these findings contribute to a growing body of evidence suggesting nutritional deficiencies play a causal role in PD. For each of the statistically significant nutraceuticals identified in this study, a biologically plausible role in neuroprotection has already been established via a wide range of mechanisms [29,30,31,32,33,34,35,36,37,38]. Experimentally, these findings provide population-based evidence that supplementation is associated with improved outcomes. The use of a remotely acquired, patient-centered outcome measure PRO-PD in this study was innovative. Due to its subjective nature, the PRO-PD is especially useful in early PD, when there are fewer motor symptoms. These data suggest the PRO-PD is an apt tool for assessing clinical manifestations associated with nutritional deficiencies and responsiveness to intervention. Observational studies provide a unique view into population trends and the pragmatic study design results in findings immediately useful to the community.

Despite the strengths of this study and the congruence between the findings presented here and prior research, this study has several limitations. This study is composed of a self-selected cohort of individuals electing to participate in a study related to lifestyle modification; this cohort may be using supplements differently from the general population. As is commonly the case in PD clinical trials, the sample population in this study was biased towards white, female individuals with higher income and education, making generalizability difficult. This cross-sectional analysis does not permit causal inference and it is evident that there is a lot of overlap between individuals taking different supplements. Future research should attempt to enroll a more heterogeneous cohort to improve the generalizability of data in future studies. Another limitation of this dataset is that some of the supplements were only being used by a small number of individuals, such as NAD+ (*n* = 19, 1.8%) and Ginkgo biloba (*n* = 16, 1.5%); statistical significance in these smaller samples should be interpreted with caution as skew and kurtosis can increase statistical significance inappropriately. This observational study has inherent weaknesses in the ability to control for quality of dosing, active ingredient quality, and regularity of supplementation from recall bias and the self-reported nature of this study. In addition, nutraceutical brands are unregulated and therefore pose weaknesses in the ability to accurately determine the true effect of the nutraceutical. These data should be interpreted with these inherent weaknesses in mind [39].

## 5. Conclusions

In this real-world natural history study, 13 of the 43 supplements studied were associated with fewer patient-reported symptoms over time, each of these with evidence of biological plausibility and an existing body of evidence supporting their use. While the supplements that were not associated with clinical benefit may not be effective at slowing progression, it is possible that the study was underpowered to detect improvement in some of these products. This study was not designed to evaluate symptom specific improvements, such as probiotics for constipation. 

Results from this natural history study suggest that some over-the-counter nutraceuticals may have therapeutic benefits. The supplements on this list with the most significant perceived impact on a population level and for which there is a biologically plausible rationale for their use in PD should be considered for evaluation in prospective intervention studies. Subsequent research on these nutraceuticals should strive to include PROs and other subjective measures of early PD that may be more sensitive than traditional objective, motor-based evaluations. Further human research on the nutraceuticals highlighted here is warranted in the search for therapeutics capable of improving PD outcomes over time. 

## Figures and Tables

**Table 1:** Descriptive statistics of the study cohort with *n* and sample percent.

	Idiopathic PD *n* = 1084
**Mean age (SD)**	66.6 (8.62)
**Years since diagnosis (SD)**	7.8 (5.41)
**Gender**	
Male	400 (36.9%)
Female	593 (54.7%)
Other/Not answered	91 (8.4%)
**Hoen & Yahr Stage**	
1: unilateral only	441 (40.7%)
2: bilateral, good balance	252 (23.2%)
3: postural instability	275 (25.4%)
4: severe disability, independent	56 (5.2%)
5: wheelchair/bed, dependent	7 (0.6%)
Don’t know/unspecified	53 (4.9%)
**Income**	
<$20K	46 (4.2%)
$20–40K	80 (7.4%)
$40–60K	140 (12.9%)
$60–80K	161 (14.9%)
$80–100K	126 (11.6%)
$100–150K	167 (15.4%)
>$150K	208 (19.2%)
Not answered	156 (14.4%)
**Education**	
Less than 8th grade	1 (0.1%)
Grades 9–11	6 (0.6%)
Completed high school/GED	88 (8.1%)
Technical school	49 (4.5%)
Associate degree	67 (6.2%)
Bachelor’s degree	272 (25.1%)
Graduate/Professional degree	507 (46.8%)
Not answered	94 (8.7%)
**Race/Ethnicity**	
Caucasian	306 (91.6%)
Black	3 (0.9%)
Hispanic	5 (1.5%)
Native American	1 (0.3%)
Asian/Pacific Islander	11 (3.3%)
Other	3 (0.9%)
N/A	5 (1.5%)

## Data Availability

Data can be requested via https://redcap.bastyr.edu/redcap/surveys/?s=MTND8FM4XY (accessed on 2 December 2022).
